# Urodynamic characterization in children with lower urinary tract symptoms and comorbid ADHD: a retrospective matched case-control study

**DOI:** 10.3389/fped.2025.1755954

**Published:** 2026-01-15

**Authors:** Ping Bai, Lili Liu, Diyi Luo

**Affiliations:** 1 Department of Pediatric Urinary Disease Center Nursing, West China Second University Hospital, Sichuan University, Chengdu, China; 2 Key Laboratory of Birth Defects and Related Diseases of Women and Children (Sichuan University), Ministry of Education, Chengdu, China

**Keywords:** ADHD, bladder capacity, case–control study, detrusor overactivity, LUTS, urodynamics

## Abstract

**Background:**

Lower urinary tract symptoms (LUTS) are common functional urinary disorders in children and can markedly impair quality of life. Attention-deficit/hyperactivity disorder (ADHD) is a prevalent neurodevelopmental condition, and emerging evidence suggests that affected children are at increased risk of LUTS. Nevertheless, systematic investigations into the relationship between ADHD and urodynamic characteristics in pediatric LUTS remain limited. This study aimed to evaluate the association between ADHD and urodynamic features in children with LUTS using a case–control design.

**Methods:**

We conducted a retrospective case–control study including 144 children with LUTS, of whom 36 were diagnosed with ADHD. All participants underwent standardized urodynamic testing, with assessments of bladder capacity, detrusor pressure at maximum filling, and detrusor overactivity (DO). Children were categorized into ADHD and non-ADHD groups, and intergroup comparisons of urodynamic parameters were performed. Multivariable regression analysis was used to assess the independent association between ADHD and urodynamic abnormalities.

**Results:**

Compared with the non-ADHD group (*n* = 108), children with ADHD (*n* = 36) exhibited significantly reduced volumes at first urge, strong urge, and maximum cystometric capacity (all *p* < 0.05). Conversely, detrusor pressure at maximum filling and the prevalence of DO were significantly higher in the ADHD group (both *p* < 0.05). Multivariable regression analysis identified DO as an independent predictor of urodynamic abnormalities in children with ADHD (OR = 3.43, 95% CI: 1.32–8.91, *p* = 0.012).

**Conclusions:**

Children with ADHD display significant functional bladder abnormalities on urodynamic testing, particularly reduced bladder capacity, increased detrusor pressure during filling, and heightened detrusor activity. ADHD may influence bladder function, at least in part, through neurobehavioral mechanisms. These findings provide valuable clinical insights for the management of LUTS in children with ADHD and underscore the importance of early screening and intervention. Further research is needed to elucidate the underlying mechanisms and to develop effective therapeutic strategies.

## 1 Background

Lower urinary tract symptoms (LUTS) are common functional voiding disorders in children, characterized by urinary frequency, urgency, daytime incontinence, voiding difficulty, and nocturnal enuresis (1). LUTS not only adversely affect children's physical and psychological well-being and social functioning but also impose a substantial burden on family quality of life (2). In recent years, increasing attention has been directed toward the association between voiding dysfunction and behavioral problems in children. Accumulating evidence suggests that LUTS frequently co-occur with neuropsychological disorders, particularly attention-deficit/hyperactivity disorder (ADHD) (3). ADHD is one of the most prevalent neurodevelopmental disorders in childhood, characterized by inattention, hyperactivity, and impulsivity, with an estimated prevalence of 5%–8% (4). Previous studies have demonstrated that children with ADHD are more likely than typically developing peers to experience urinary symptoms, with a significantly higher prevalence of comorbid LUTS. Proposed mechanisms include central nervous system dysregulation, impaired executive control, and altered processing of somatosensory signals involved in bladder function (5). However, most existing studies rely primarily on questionnaires and symptom rating scales, whereas objective and systematic evaluations of bladder function in children with ADHD remain scarce.

Urodynamic testing, regarded as the gold standard for assessing bladder storage and voiding function, provides objective parameters such as bladder capacity, detrusor activity, bladder compliance, and post-void residual (PVR) (6). To date, direct urodynamic comparisons between children with LUTS with and without comorbid ADHD are limited. Therefore, the present study employed a retrospective matched case–control design to objectively compare urodynamic characteristics between children with LUTS with and without comorbid ADHD, aiming to clarify the relationship between ADHD and specific bladder dysfunctions and to provide objective, clinically relevant evidence for patient management and mechanistic research.

## 2 Methods

### 2.1 Study design and participants

This single-center, retrospective matched case–control study was conducted at the Department of Pediatric Nephrology, West China Second University Hospital, Sichuan University. Medical records of children presenting with LUTS who underwent urodynamic testing between January 2024 and December 2024 were reviewed. The case group consisted of children diagnosed with ADHD, with diagnoses established by qualified pediatric neurologists or psychiatrists according to the Diagnostic and Statistical Manual of Mental Disorders, Fifth Edition (DSM-5). The control group comprised children with LUTS but without ADHD, matched to cases by age (±1 year) and sex at a ratio of 1:3.

In our institution, urodynamic testing is not performed as a first-line diagnostic procedure. Consistent with the recommendations of the International Children's Continence Society (ICCS), invasive multichannel urodynamic studies were reserved for children with persistent or complex LUTS after non-invasive evaluation and initial conservative management. All included children underwent initial conservative treatment prior to referral for urodynamic evaluation. First-line management typically consisted of standard urotherapy, including behavioral modification, timed voiding, and fluid management, with or without pharmacological therapy when clinically indicated. Urodynamic studies were performed only in children with refractory symptoms despite initial management or when underlying bladder dysfunction was clinically suspected.

This study was approved by the Ethics Committee of West China Second University Hospital, Sichuan University (Approval number: Medical Research 2025 Approval No.461). Given the retrospective design and anonymized data extraction, the requirement for informed consent was waived. All procedures adhered to the principles of the Declaration of Helsinki. Urodynamic studies were performed only after confirming the absence of urinary tract infection, verified by a negative urine culture before the examination.

### 2.2 Inclusion and exclusion criteria

**Inclusion criteria**: Children were eligible if they met all of the following: (1) aged 5–17 years; (2) clinically diagnosed with functional LUTS in accordance with the International Children's Continence Society (ICCS) criteria; (3) completion of standardized urodynamic testing with complete reports available; and (4) for the case group, a confirmed ADHD diagnosis established by a pediatric neurologist or psychiatrist based on DSM-5 criteria.

**Exclusion criteria:** Children were excluded if any of the following applied: (1) concomitant organic neurological disorders (e.g., spina bifida, cerebral palsy), genitourinary anatomical abnormalities, or neurogenic bladder; (2) history of urologic surgery; (3) intellectual disability or severe cognitive impairment; (4) comorbid psychiatric or behavioral disorders [e.g., autism spectrum disorder (ASD), oppositional defiant disorder (ODD)]; (5) use of medications that could affect bladder function within two weeks prior to testing; or (6) incomplete clinical records or urodynamic data.

### 2.3 Urodynamic assessment

All participants underwent multichannel urodynamic studies in accordance with ICCS standards. Examinations were performed by experienced technicians using a multichannel urodynamic system (model GBS002, Laborie, Canada). The assessment included: (1) **Filling phase evaluation:** bladder sensation, compliance, detrusor overactivity (DO), and maximum cystometric capacity (MCC); (2) **Pressure–flow evaluation:** detrusor pressure at maximum flow (Pdet.Qmax), maximum flow rate (Qmax), voided volume, voiding time, and time to maximum flow (time to Qmax). The filling rate was standardized at 10% of the estimated bladder capacity per minute. All urodynamic traces and measurements were interpreted by a pediatric nephrologist blinded to participants' ADHD status to minimize observer bias.

### 2.4 Data collection and variable definitions

Demographic characteristics (age and sex), clinical information, and urodynamic parameters were extracted from medical records. Urodynamic variables included bladder sensation thresholds (volumes at first sensation, first desire to void, and strong desire to void), MCC, DO, maximum detrusor pressure during the filling phase, Pdet.Qmax, Qmax, PVR and bladder contractility index (BCI). DO was defined as any involuntary detrusor contraction occurring during the filling phase with an amplitude greater than 15 cmH_2_O, in accordance with ICCS recommendations. BCI was calculated using the standard formula: BCI = Pdet.Qmax + 5 ×  Qmax.

### 2.5 Statistical analysis

Normality of continuous variables was assessed using the Shapiro–Wilk test, which indicated that all urodynamic parameters were non-normally distributed. Continuous variables were therefore summarized as medians with interquartile ranges (IQRs). Between-group comparisons were performed using the Wilcoxon rank-sum test for continuous variables and the McNemar test or Fisher's exact test for categorical variables. To further evaluate the independent association between ADHD and urodynamic parameters, conditional logistic regression models were constructed, with covariates identified as potential sources of selection bias in preliminary analyses included for adjustment. Model discrimination was assessed using receiver operating characteristic (ROC) curve analysis, and predictive performance was quantified by calculating the area under the curve (AUC).

All statistical tests were two-tailed, with *p* < 0.05 considered statistically significant. Analyses were performed using SPSS version 27.0 (IBM Corp., Armonk, NY, USA) and R software version 4.5.1 (R Foundation for Statistical Computing, Vienna, Austria).

## 3 Results

### 3.1 Baseline characteristics and univariable analysis

A total of 284 children met the eligibility criteria, of whom 36 were included in the ADHD group. Using a 1:3 matching ratio, 108 controls without ADHD were selected. As age and sex were identical between the two groups by design, no statistical testing was performed for these variables. Univariable analyses of urodynamic parameters are summarized in Table 1.

**Table 1:** Clinical presentation of LUTS and univariate comparison of urodynamic parameters between ADHD patients and matched controls.

Variable	ADHD patients (*n* = 36)	Controls (*n* = 108)	*p*
Age (years)^a^	8.0 (6.0–9.0)	8.0 (6.0–9.0)	—
Gender^b^, male	26 (72.2%)	78 (72.2%)	—
LUTS^c^			
Nocturnal enuresis ^b^	27 (75.0%)	87 (80.6%)	0.484
Daytime incontinence^b^	4 (11.1%)	9 (8.3%)	0.737
Urinary frequency^b^	5 (13.9%)	12 (11.1%)	0.766
Maximum detrusor pressure during filling (cmH_2_O)^a^	24.0 (16.0–56.8)	15.0 (9.3–33.0)	0.004
First sensation (mL)^a^	75.5 (48.3–113.5)	86.5 (50.5–131.8)	0.249
First urge (mL)^a^	98.0 (85.0–165.5)	143.0 (97.3–186.0)	0.018
Strong urge (mL)^a^	131.0 (93.5–189.0)	163.5 (119.0–202.5)	0.018
MCC (mL)^a^	141.6 (95.8–193.0)	178.0 (129.9–216.0)	0.022
Qmax (mL/s)^a^	5.9 (4.1–8.3)	6.1 (4.0–10.0)	0.888
Pdet.Qmax (cmH_2_O)^a^	51.4 (40.8–82.2)	49.4 (35.0–72.5)	0.201
DO (yes)^b^	26 (72.2%)	44 (40.7%)	<0.001
BCI^a^	100.5 (75.0–121.0)	84.5 (66.3–110.5)	0.117

Age and sex were used as matching variables and were therefore not subjected to statistical significance testing.

LUTS, lower urinary tract symptoms; Urinary frequency refers to an increased number of voids during daytime and/or nighttime compared with normal expectation for age; MCC, maximum cystometric capacity; Qmax, maximum flow rate; Pdet.Qmax, detrusor pressure at maximum flow; DO, detrusor overactivity; BCI, bladder contractility index.

a Median (IQR).

b Percentiles (%).

c Symptoms represent the child's predominant LUTS at presentation, recorded as part of the clinical profile rather than as standalone indications for urodynamic testing.

With respect to lower urinary tract symptoms, no significant differences were found between the ADHD and control groups in nocturnal enuresis, daytime incontinence, or urinary frequency (*p* = 0.484, 0.737, and 0.766, respectively). However, in storage-phase parameters, children with ADHD exhibited significantly higher maximum detrusor pressure during filling (24.0 vs. 15.0 cmH₂O, *p* = 0.004). Moreover, bladder capacity–related measures were consistently reduced in the ADHD group, including volumes at first urge (98.0 vs. 143.0 mL, *p* = 0.018), strong urge (131.0 vs. 163.5 mL, *p* = 0.018), and MCC (141.6 vs. 178.0 mL, *p* = 0.022). The prevalence of DO was also markedly higher in the ADHD group compared with controls (72.2% vs. 40.7%, *p* < 0.001).

By contrast, no significant group differences were observed in voiding-phase parameters, including Qmax, Pdet.Qmax, and BCI (*p* > 0.05 for all). Collectively, these findings indicate that storage-phase dysfunction, characterized by increased DO, elevated filling-phase detrusor pressure, and reduced bladder capacity, is more pronounced in children with ADHD.

#### 3.1.1 Comparison of matched versus unmatched controls

To examine the potential impact of selection bias, urodynamic parameters were compared between matched (*n* = 108) and unmatched (*n* = 140) controls, yielding a total of 248 control cases. As shown in Supplementary Table S1, significant differences were observed in age, sex, Qmax, and detrusor pressure at Pdet.Qmax (*p* < 0.05). These discrepancies suggest the presence of selection bias, which may influence the external generalizability of the study findings.

### 3.2 Multivariable conditional logistic regression analysis

To further evaluate the independent association between ADHD and urodynamic parameters while accounting for potential selection bias and confounding factors, a multivariable conditional logistic regression analysis was performed. Variables with clinical relevance or a *P* value < 0.05 in the univariable analysis were included in the model, namely the presence of involuntary detrusor contractions, maximum detrusor pressure during filling, bladder sensation thresholds (first urge and strong urge), MCC, age, sex, Qmax, and Pdet.Qmax (see Table 2).

**Table 2:** Multivariate conditional logistic regression analysis of urodynamic parameters associated with ADHD.

Variable	***β*** (Regression coefficient)	OR Value	Confidence Interval (Lower, Upper)	*p*
Age(years)	0.040	1.05	(0.82, 1.33)	0.723
Gender, male	−0.370	0.69	(0.26, 1.83)	0.458
Maximum detrusor pressure during filling (cmH_2_O)	−0.004	1.00	(0.99, 1.01)	0.819
First urge (mL)	−0.002	1.00	(0.98, 1.01)	0.681
Strong urge (mL)	−0.001	1.00	(0.99, 1.01)	0.771
MCC (mL)	−0.001	1.00	(0.99, 1.01)	0.960
Qmax (mL/s)	−0.018	0.98	(0.93, 1.04)	0.556
Pdet.Qmax (cmH_2_O)	0.010	1.01	(0.99, 1.02)	0.442
**DO (yes)**	**1** **.** **232**	**3** **.** **43**	**(1.32, 8.91)**	**0** **.** **012**

Conditional logistic regression was performed based on age- and sex-matched sets.

*β* values represent regression coefficients from the multivariable model.

Bold values indicate statistical significance (*p* < 0.05).

DO, detrusor overactivity; MCC, maximum cystometric capacity; Qmax, maximum flow rate; Pdet.Qmax, detrusor pressure at maximum flow.

Regression analysis identified the presence of involuntary detrusor contractions as an independent predictor of ADHD in children (OR = 3.43, 95% CI: 1.32–8.91, *p* = 0.012). This finding indicates that children with ADHD are more than three times as likely to exhibit detrusor overactivity compared with non-ADHD peers. Other variables did not retain statistical significance after adjustment for covariates (*p* > 0.05), suggesting that although group differences were observed in the univariable analyses, these factors were not independently associated with ADHD. The model demonstrated good fit, stable standard errors for regression coefficients, and no evidence of multicollinearity, supporting the robustness of the analysis.

### 3.3 Model discrimination analysis

**Figure 1:**
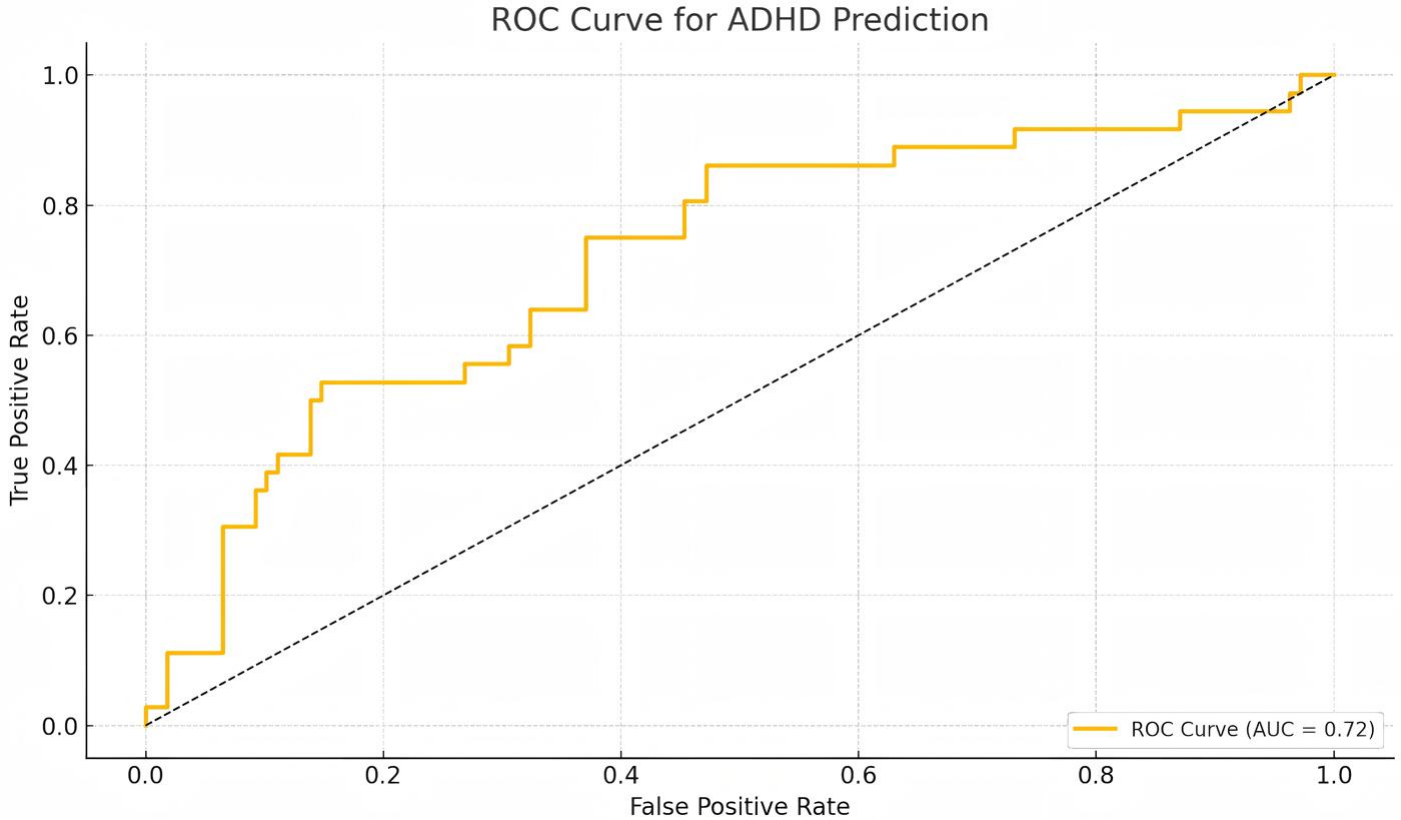
Receiver operating characteristic (ROC) curve for the prediction of attention-deficit/hyperactivity disorder (ADHD). The true positive rate (sensitivity) is plotted against the false positive rate (1 – specificity). The dashed diagonal line represents random classification. The area under the curve (AUC) is 0.72, indicating moderate discriminative performance.

To evaluate the discriminative performance of the multivariable conditional logistic regression model, a receiver operating characteristic (ROC) curve was constructed (see Figure 1). The analysis demonstrated an area under the curve (AUC) of 0.72, indicating moderate discriminative ability. The ROC curve was consistently positioned above the reference line (diagonal), suggesting that the model could moderately discriminate children with ADHD based on urodynamic characteristics. These findings further support the robustness of the regression model and highlight its potential clinical utility.

## 4 Discussion

In this retrospective matched case–control study, we investigated the association between ADHD and urodynamic characteristics in children with LUTS. Our findings revealed significant differences in multiple urodynamic parameters between children with and without ADHD, particularly in bladder sensory thresholds, MCC, and detrusor pressure and activity, highlighting distinct patterns of bladder dysfunction in children with ADHD.

Children with ADHD exhibited significantly higher maximum detrusor pressure during the filling phase, suggesting elevated intravesical pressure and impaired bladder storage function. In parallel, MCC was markedly reduced, indicating decreased bladder compliance and storage capacity. Sensory volumes at first sensation and strong desire to void were also significantly lower in the ADHD group, reflecting altered bladder sensation, which may be related to central nervous system dysregulation. Notably, the prevalence of DO was substantially higher in children with ADHD (72.2% vs. 40.7%), further supporting a close association between ADHD and objective urodynamic abnormalities. These findings are consistent with previous international studies reporting a higher burden of voiding dysfunction in children with ADHD, particularly nocturnal enuresis and other storage-related symptoms (7–9). Moreover, established risk factors for enuresis in children with ADHD—including male sex, lower parental education, neonatal factors, family history of enuresis, low birth weight, and cesarean delivery—further suggest that ADHD may influence bladder sensory regulation and storage function (9). An epidemiological survey of rural Chinese children also indicated that functional voiding disorders may serve as early clinical markers of ADHD, underscoring the importance of heightened awareness and early screening, especially in resource-limited settings (10).

The neurobiological characteristics of ADHD provide a plausible framework for understanding its association with bladder dysfunction. ADHD is a highly prevalent neurodevelopmental disorder affecting more than 5% of children and adolescents worldwide (11). Core pathophysiological features include dysfunction of the prefrontal cortex, a region essential for inhibitory control and sensory integration, both of which are critical for normal bladder regulation. Impaired prefrontal modulation may weaken voluntary control over the micturition reflex, predisposing individuals to involuntary detrusor contractions during bladder filling. Under physiological conditions, the detrusor muscle remains relaxed during filling; in contrast, DO leads to increased bladder pressure and may manifest clinically as urgency or voiding difficulty. In addition, deficits in self-regulation and impulse control commonly observed in ADHD may further disrupt coordinated bladder storage and voiding. Neurotransmitter dysregulation, particularly involving norepinephrine and serotonin, has also been implicated in both ADHD and lower urinary tract control, potentially contributing to abnormal bladder sensation and voiding behavior. A recent systematic review and meta-analysis emphasized the clinical relevance of recognizing the association between LUTS and ADHD, particularly for pediatricians, urologists, and child psychiatrists, to facilitate comprehensive assessment and optimize management strategies (3).

To assess the independent association between ADHD and urodynamic abnormalities, we performed multivariable conditional logistic regression analyses adjusting for age and sex. Detrusor overactivity emerged as a significant independent factor associated with ADHD (OR = 3.43, 95% CI: 1.32–8.91, *p* = 0.012), consistent with previous studies (8, 13, 14) children with ADHD were more than three times as likely to exhibit DO compared with non-ADHD peers. Although several urodynamic parameters differed between groups in univariable analyses, these associations did not persist after multivariable adjustment, which may reflect limited statistical power, inter-individual variability, or the absence of independent effects of specific measures.

ROC curve analysis demonstrated that the multivariable model achieved an AUC of 0.72, indicating moderate discriminative performance. While this level of accuracy does not support the use of the model as a standalone diagnostic tool, it provides clinically relevant information that may aid in risk stratification and preliminary screening. Future studies incorporating additional clinical variables, such as neuropsychological assessments, behavioral measures, or imaging biomarkers, may further enhance predictive performance and improve clinical applicability.

## 5 Conclusion

This retrospective matched case–control study provides objective urodynamic evidence demonstrating a significant association between ADHD and bladder dysfunction in children with LUTS. Children with ADHD exhibited distinct urodynamic abnormalities, including reduced bladder capacity, altered bladder sensory thresholds, and a markedly higher prevalence of detrusor overactivity.

Multivariable analysis identified detrusor overactivity as an independent urodynamic factor associated with ADHD, highlighting a potential link between neurobehavioral dysregulation and impaired bladder storage function. Although the discriminative performance of the predictive model was moderate, the findings underscore the clinical relevance of urodynamic evaluation in selected children with complex or refractory LUTS and comorbid ADHD.

Overall, this study emphasizes the importance of considering underlying bladder dysfunction in children with ADHD and LUTS and provides objective evidence to support more comprehensive clinical assessment and individualized management strategies.

## Data Availability

The original contributions presented in the study are included in the article/Supplementary Material, further inquiries can be directed to the corresponding author.
