# Supplementary material for: Urodynamic characterization in children with lower urinary tract symptoms and comorbid ADHD: a retrospective matched case-control study
Source: Front Pediatr. 2026 Jan 15;13:1755954. doi: 10.3389/fped.2025.1755954 (PMC12852382; doi:10.3389/fped.2025.1755954)
Supplement: Supplementary file 1 [file Table1.docx]

**Table S1. Comparison of urodynamic parameters between matched and unmatched control groups (median [IQR])**

| **Variable** | **Matched Controls**  **(n = 108)** | **Unmatched Controls**  **(n = 140)** | ***P*** |
| --- | --- | --- | --- |
| Age(years) | 8.0(6.0-9.0) | 9.0（7.0-10.0） | 0.004 |
| Gender ^b^, male | 78(72.2%) | 30(21.4%) | ＜0.001 |
| Maximum detrusor pressure during filling（cmH_2_O）^a^ | 15.0(9.3-33.0) | 15.0(9.0-28.0) | 0.526 |
| First sensation（ml）^a^ | 86.5（50.5-131.8） | 87.5(55.0-120.8) | 0.712 |
| First urge（ml）^a^ | 143.0(97.3-186.0) | 140.5(103.3-194.8) | 0.732 |
| Strong urge（ml）^a^ | 163.5（119-202.5） | 166.0(123.0-220.8) | 0.683 |
| MCC（ml）^a^ | 178.0(129.9-216.0) | 175.3（128.7-226.1） | 0.740 |
| Qmax（ml/s）^a^ | 6.1(4.0-10.0) | 8.3（5.0-13.0） | 0.003 |
| Pdet.Qmax（cmH_2_O）^a^ | 49.4(35.0-72.5) | 42.8(31.4-57.9) | 0.040 |
| DO (yes)^b^ | 44（40.7%） | 55（39.3%） | 0.896 |
| BCI ^a^ | 84.5(66.3-110.5) | 91.0(70.0-118.0) | 0.215 |

a Median (IQR); b Percentiles(%)
